# Heritability of siesta and night-time sleep as continuously assessed by a circadian-related integrated measure

**DOI:** 10.1038/s41598-017-12460-x

**Published:** 2017-09-26

**Authors:** J. Lopez-Minguez, J. J. Morosoli, J. A. Madrid, M. Garaulet, J. R. Ordoñana

**Affiliations:** 1 Department of Physiology Faculty of Biology, University of Murcia, Murcia, Spain; 2 IMIB-Arrixaca, Murcia, Spain; 3 Department of Human Anatomy and Psychobiology, School of Psychology, University of Murcia, Murcia, Spain

## Abstract

Siesta is a relevant aspect of sleep due to its posited relationship with health or cognitive function. However, unlike night-time sleep, studies about daytime-sleep determinants and characteristics are scarce, and the genetic/environmental structure of siesta is still unknown. Our aim was to explore the relative contribution of genetic and environmental factors to variation in sleep-wake rhythm, measured by a continuous assessment of temperature-activity-position (TAP), which allows for diurnal sleep analysis. The sample comprised 53 pairs of female twins (28 MZ and 25 DZ), selected from the Murcia Twin Register. Mean age of participants was 52 (SD: 6.03). Zygosity was determined by DNA. We conducted separate univariate analyses to study the sources of variance of daytime and night-time sleep parameters. About 60% of the sample reported to take siesta at least once a week. Heritability of taking siesta and daytime sleep duration was 65 and 61% respectively. Other sleep parameters obtained by TAP showed heritability estimates between 36 and 69%, suggesting a relevant impact of genetic factors on sleep rhythm. This is the first study to investigate the relative contribution of genetic factors to siesta. By using TAP, we introduce a novel approach to the study of diurnal sleep characteristics.

## Introduction

Siesta may be defined as a regular afternoon sleep or midday nap characteristic of some countries and areas of the world such as the Mediterranean countries, China, and Latin America^1^. This practice is important to consider due to its possible relationship with relevant aspects of human life, such as health or cognitive functioning^2,3^. In the past few years, midday nap has become a “hot topic” since previous studies suggested that taking a short siesta could be beneficial, particularly in some specific populations, such as shift workers^4,5^. Short siesta (less than 30 minutes) appears to reduce blood pressure and decrease the prevalence of hypertension^6^. By contrast, several authors have speculated about siesta being a marker of unhealthy conditions, probably related to some diseases, such as diabetes^7^, Parkinson^8^, myocardial infarction^9^ and obesity^10^. Nonetheless, it is unclear if siesta is the cause or the consequence of these health problems.

Siesta habits show a geographical distribution, and have been traditionally considered as a behaviour that depends on culture and environmental conditions. However, recent genome-wide association analyses of sleep disturbance traits have identified new loci related not only to night-time sleep duration and insomnia, but also to daytime sleepiness^11^. This suggests a relative role of genetic factors in siesta habits and its characteristics. Nonetheless, there are to our knowledge no studies about the relative contribution of genetic and environmental factors to siesta assessed by objective data of diurnal sleep.

Twin and family studies have provided estimates of heritability for a number of sleep related variables^12^. In this context, significant heritabilities have been reported for sleep duration (39–44%)^13,14^ or different measures of sleep quality (33–46%)^15,16^. Other indexes of circadian rhythmicity related to sleep, such as the morning-evening questionnaire (MEQ) scores have yielded similar estimates between 44% and 47%^17^. In fact, several approaches have been used to study the relative role of genetics and environment in sleep through a) subjective methods, which imply the use of questionnaires to obtain sleep characteristics^15^; b) objective methods, which provide greater insights about the physiological aspects of sleep, such as the examination of brain activity patterns using polysomnography (PSG) or electroencephalography (EEG)^18,19^. Nevertheless, these objective techniques tend to focus exclusively on nocturnal sleep, excluding daytime sleep episodes as siesta. Furthermore, these techniques depend on an artificial and time-limited environment, and they don’t allow to continuously record for long periods of time, which is crucial for studying the various characteristics of the siesta pattern, such as frequency, length and type. In order to assess an accurate pattern, and given that the analysis is in healthy individuals without pathological alterations in the sleep rhythm, the 7 complete and consecutive day record is enough for the evaluation of their pattern^20,21^. PSG could also interfere with the subject’s routine and affect the activity-rest behaviour. It is thus essential to use a different technique, in order to avoid these problems and to allow for correct and comfortable ambulatory measures through the use of external sensors.

Integrative variables, such as TAP (temperature, activity and position), can be helpful to obtain circadian patterns and rhythmic parameters^22,23^. Use of TAP-derived sleep patterns has been validated as an appropriate measure for the study of sleep by its association to PSG. Furthermore, it has been considered a useful tool for preliminary screening of subjects suspected to have sleep problems and to detect sleep pathologies^24^.

TAP has not yet been used to investigate the relative contribution of genetics to daytime sleep patterns. The aim of this study is to explore the genetic and environmental underpinnings of variations in the sleep-wake rhythm using TAP, including siesta and night-time sleep.

## Results

General characteristics of the studied population are presented in Table 1. About 60% of the total sample took a siesta at least once a week. The average duration of daytime sleep, objectively measured, was approximately 45 minutes. With respect to nocturnal sleep, average duration was about 7 hours. The results of MEQ indicate that our sample tended to be neither-type (score = 42–58).

**Table 1:** General characteristics of the sample.

	Monozygotic (n = 56)	Dizygotic (n = 50)	*p* values
**Age (y.)**	51 ± 6	53 ± 6	0.066
BMI (kg/m^2^)	26.30 ± 3.89	25.66 ± 3.65	0.404
**Daytime sleep characteristics**			
Take siesta (Self-reported) (%)	63%	60%	0.540
Self-reported siesta duration (mm)	51 ± 23	49 ± 26	0.744
TAP-measured daytime sleep duration (mm)	49.3 ± 37.3	41.1 ± 31.6	0.296
**Night-time sleep characteristics**			
Self-reported sleep duration (hh:mm)	6:32 ± 0:52	6:47 ± 1:04	0.159
TAP-measured night-time sleep duration (hh:mm)	7:08 ± 0.53	7:13 ± 0:55	0.691
**Morning-evening questionnaire (score)**	55.21 ± 8.67	56.44 ± 7.56	0.442

Data are represented as means ± SD. Abbreviations: BMI, body mass index; TAP, integrated measure of temperature (T), activity (A) and position (P).

**Figure 1:**
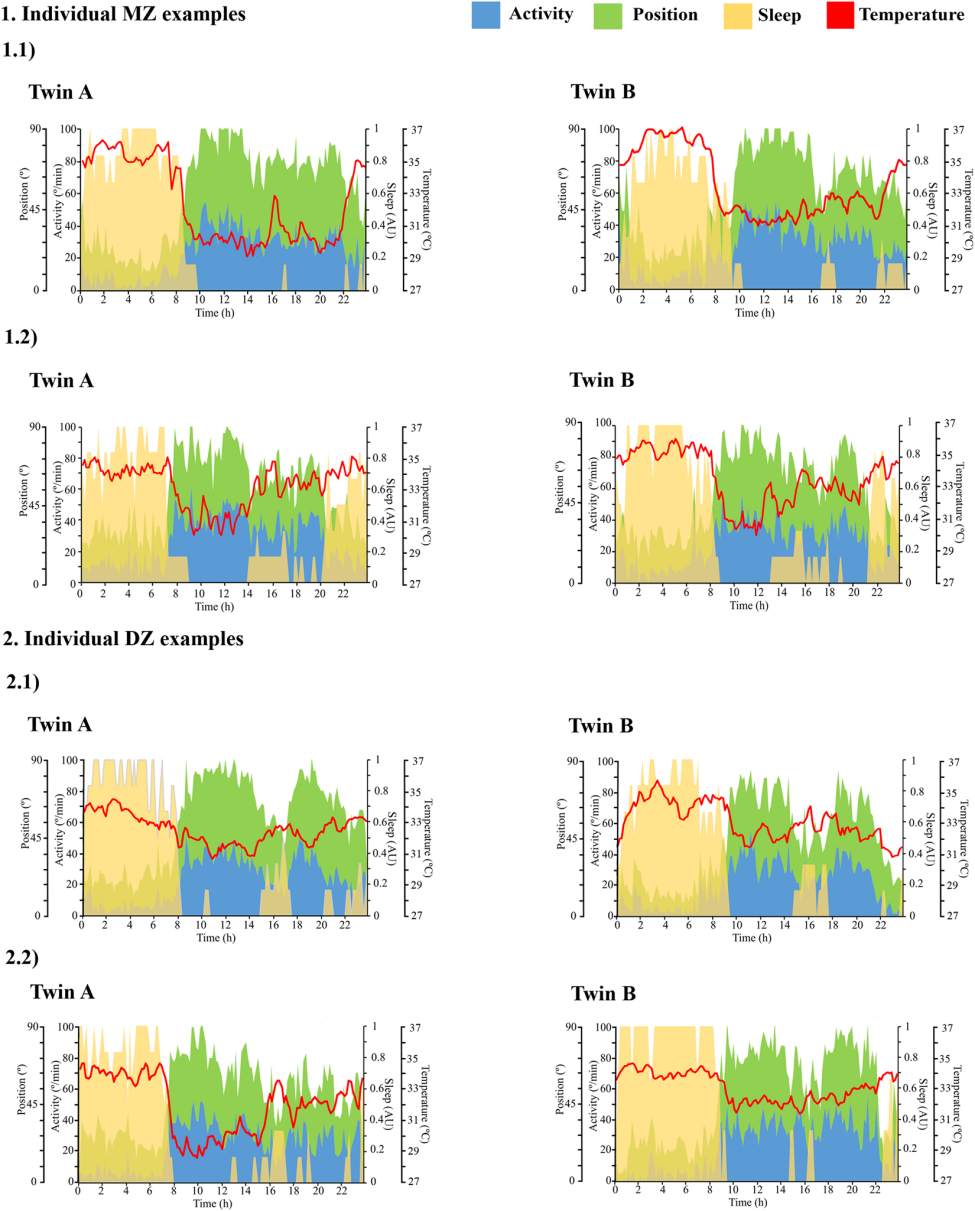
Temperature, activity, position and sleep patterns. Each graphic shows the daily patterns of one of the sisters of the pair, recorded over a 7-days period; the red line represents the temperature pattern, the blue area represents the activity pattern, the green area represents the position pattern and the yellow area represents the sleep pattern. Sleep rhythm is shown as the probability to find a subject asleep at any given time with values ranging from 0 to 1, where 1 indicates totally asleep and 0 indicates totally awake. From the total sample studied, we selected two examples from the monozygotic pairs that showed similar patterns between sisters (1.1 and 1.2) and those showing different patterns from the dizygotic pairs (2.1 and 2.2). From Kronowizard platform (https://kronowizard.um.es/).

Visual inspection of the mean waveform charts of sleep produced by TAP (Fig. 1) showed greater similarity between MZ pairs than between DZ pairs. This figure represents two MZ and two DZ twin pairs selected as examples of thepatterns. Sleep rhythm is showed as the probability to find a subject asleep at any given time with values ranging from 0 to 1, where 1 indicates totally asleep and 0 indicates totally awake. Figure 1-1.1 and 1-1.2 showed that not only night-time sleep but also day-time sleep was similar between MZ sisters, while between DZ (Fig. 1-2.1 and 1-2.2) the differences were more marked.

This greater resemblance between MZ pairs was further confirmed by the intra-class correlations, which were consistently greater for MZ than for DZ pairs (Table 2). Those correlations suggested the presence of dominant genetic effects (MZ correlations were more than twice the DZ correlations). Hence, ADE models were fitted to the data (except for relative amplitude, where an ACE model was applied). Next, nested sub-models, where D/C and A parameters were consecutively dropped, were tested against the full models to assess significance of these factors. For all variables, best-fitting models included a genetic component and residual non-shared environmental sources of variation (AE). The genetic component in the AE models could not be dropped in most cases (all but intradaily variability) since a simpler E model produced a significant worsening of fit (Table 3).

**Table 2:** Twin intra-class correlations for sleep-related parameters.

	Intra-class correlation	
	r MZ (CI 95%)	r DZ (CI 95%)
Siesta (Self-report)	0.694 (0.196, 0.932)	0.156 (−0.443, 0.672)
Daytime sleep duration	0.671 (0.379, 0.839)	−0.014 (−0.427, 0.408)
Night-time sleep duration	0.706 (0.419, 0.844)	−0.116 (−0.495, 0.314)
Mesor	0.749 (0.478, 0.884)	0.100 (−0.331, 0.495)
Acrophase	0.700 (0.387, 0.861)	0.060 (−0.349, 0.451)
Total daily sleep duration	0.730 (0.444, 0.874)	−0.124 (−0.513, 0.309)
Sleep depth (%)	0.648 (0.341, 0.827)	0.246 (−0.186, 0.594)
Percentage of rhythmicity (PR)	0.572 (0.029, 0.785)	−0.057 (−0.440, 0.345)
Interdaily stability (IS)	0.644 (0.322, 0.828)	0.028 (−0.373, 0.422)
Intradaily variability (IV)	0.436 (0.029, 0.712)	−0.117 (−0.503, 0.309)
Relative amplitude (RA)	0.655 (0.349, 0.831)	0.398 (−0.031, 0.698)
Circadian Function Index (CFI)	0.653 (0.335, 0.832)	0.040 (−0.365, 0.435)

**Table 3:** Model-fitting results for univariate models for different sleep phenotypes; and proportions of variance (parameter estimates) explained by: additive genetic influences (A), common environment (C) or dominant genetic influences (D) and residual variation (E) with 95% confidence intervals (CI).

	Goodness-of-fit index							Parameter estimates (CI = 95%)		
	Model	−2LL	df	AIC	∆χ^2^	∆df	p	A	D/C	E
*Siesta (Self-report)*	ADE	136.91	103	−69.09	—	—	—	0 (0, 0.91)	0.69 (0, 0.93)	0.31 (0.07, 0.80)
	**AE**	**137.27**	**104**	**−70.73**	**0.36**	**1**	**0.549**	**0.65 (0.17, 0.92)**	—	**0.35 (0.08, 0.83)**
	E	143.88	105	−66.12	6.97	2	0.031	—	—	1 (1, 1)
*Daytime sleep duration*	ADE	239.31	86	67.31				0 (0, 0.76)	0.64 (0, 0.80)	0.36 (0.20, 0.66)
	**AE**	**240.75**	**87**	**66.75**	**1.44**	**1**	**0.230**	**0.61 (0.29, 0.79)**	—	**0.39 (0.21, 0.71)**
	E	252.23	88	76.23	12.92	2	0.002	—	—	1 (1, 1)
*Night-time sleep duration*	ADE	223.28	85	53.28				0 (0, 0.77)	0.70 (0, 0.84)	0.30 (0.16, 0.62)
	**AE**	**226.15**	**86**	**54.15**	**2.87**	**1**	**0.090**	**0.65 (0.26, 0.84)**	—	**0.35 (0.16, 0.74)**
	E	234.95	87	60.95	16.05	2	0.001	—	—	1 (1, 1)
*Mesor*	ADE	−298.8	86	−470.80	—	—	—	0 (0, 0.82)	0.71 (0, 0.85)	0.29 (0.15, 0.56)
	**AE**	**−297,32**	**87**	**−471.32**	**1.48**	**1**	**0.224**	**0.69 (0.38, 0.85)**	—	**0.31 (0.15, 0.62)**
	E	−284.57	88	−460.57	14.23	2	0.001			1 (1, 1)
*Acrophase*	ADE	232.68	85	62.84	—			0 (0, 0.72)	0.53 (0, 0.73)	0.47 (0.27, 0.78)
	**AE**	**233.13**	**86**	**61.12**	**0.45**	**1**	**0.502**	**0.52 (0.19, 0.73)**	—	**0.48 (0.27, 0.80)**
	E	242.13	87	68.13	9.45	2	0.009			1 (1, 1)
*Total daily sleep duration*	ADE	279.15	86	107.15	—			0 (0, 0.82)	0.69 (0, 0.84)	0.31 (0.16, 0.59)
	**AE**	**280.32**	**87**	**106.32**	**1.17**	**1**	**0.279**	**0.67 (0.35, 0.84)**	—	**0.33 (0.16, 0.65)**
	E	292.54	88	116.54	13.39	2	0.001			1 (1, 1)
*Sleep depth (%)*	ADE	510.39	86	338.39	—			0 (0, 0.65)	0.53 (0, 0.74)	0.47 (0.26, 0.81)
	**AE**	**512.41**	**87**	**338.41**	**2.02**	**1**	**0.155**	**0.46 (0.11, 0.72)**	—	**0.54 (0.28, 0.89)**
	E	518.69	88	342.69	8.30	2	0.016			1 (1, 1)
*Percentage of rhythmicity (PR)*	ADE	681.39	86	509.39	—			0 (0, 0.68)	0.54 (0, 0.74)	0.46 (0.26, 0.81)
	**AE**	**682.90**	**87**	**508.90**	**1.51**	**1**	**0. 219**	**0.48 (0.12, 0.72)**	—	**0.52 (0.28, 0.88)**
	E	689.55	88	513.60	8.16	2	0.017			1 (1, 1)
*Interdaily stability (IS)*	ADE	−146.58	86	−318.58	—			0 (0, 0.74)	0.61 (0, 0.79)	0.39 (0.21, 0.72)
	**AE**	**−144.99**	**87**	**−318.99**	**1.59**	**1**	**0.207**	**0.57 (0.21, 0.78)**	—	**0.43 (0.22, 0.79)**
	E	−136.28	88	−312.28	10.30	2	0.006			1 (1, 1)
*Intradaily variability (IV)*	ADE	−168.00	86	−340.00	—			0 (0, 0.63)	0.47 (0, 0.73)	0.53 (0.27, 1)
	**AE**	**−166.54**	**87**	**−340.54**	**0.00**	**1**	**0.227**	**0.36 (0, 0.68)**	—	**0.64 (0.31, 1)**
	E	−164.05	88	−340.05	11.86	2	0.139			1 (1, 1)
*Relative amplitude (RA)*	ACE	232.28	86	60.28				0.47 (0, 0.79)	0.15 (0, 0.67)	0.37 (0.20, 0.67)
	**AE**	**232.42**	**87**	**58.42**	**0.14**	**1**	**0.708**	**0.63 (0.36, 0.80)**	—	**0.37 (0.20, 0.64)**
	CE	233.65	87	59.65	1.37	1	0.242		0.52 (0.27, 0.71)	0.48 (0.29, 0.73)
	E	247.04	88	71.04	14.76	2	0.001			1 (1, 1)
*Circadian Function Index (CFI)*	ADE	−235.99	86	−407.99	—			0 (0, 0.75)	0.63 (0, 0.80)	0.37 (0.20, 0.70)
	**AE**	**−234.39**	**87**	**−408.39**	**1.60**	**1**	**0.206**	**0.58 (0.23, 0.79)**	—	**0.42 (0.21, 0.77)**
	E	−224.96	88	−400.96	11.03	2	0.004			1 (1, 1)

*−2LL*: twice negative log-likelihood; *df*: degrees of freedom; AIC: Akaike Information Criterion; ∆χ^2^: difference in ﻿χ^﻿2^ to full model; ∆*df*: difference in degrees of freedom to full model. Bold values indicate best fitting model. Since DZ correlation was greater than half of the MZ correlation, an ACE model was fitted for *relative amplitude*.

**Figure 2:**
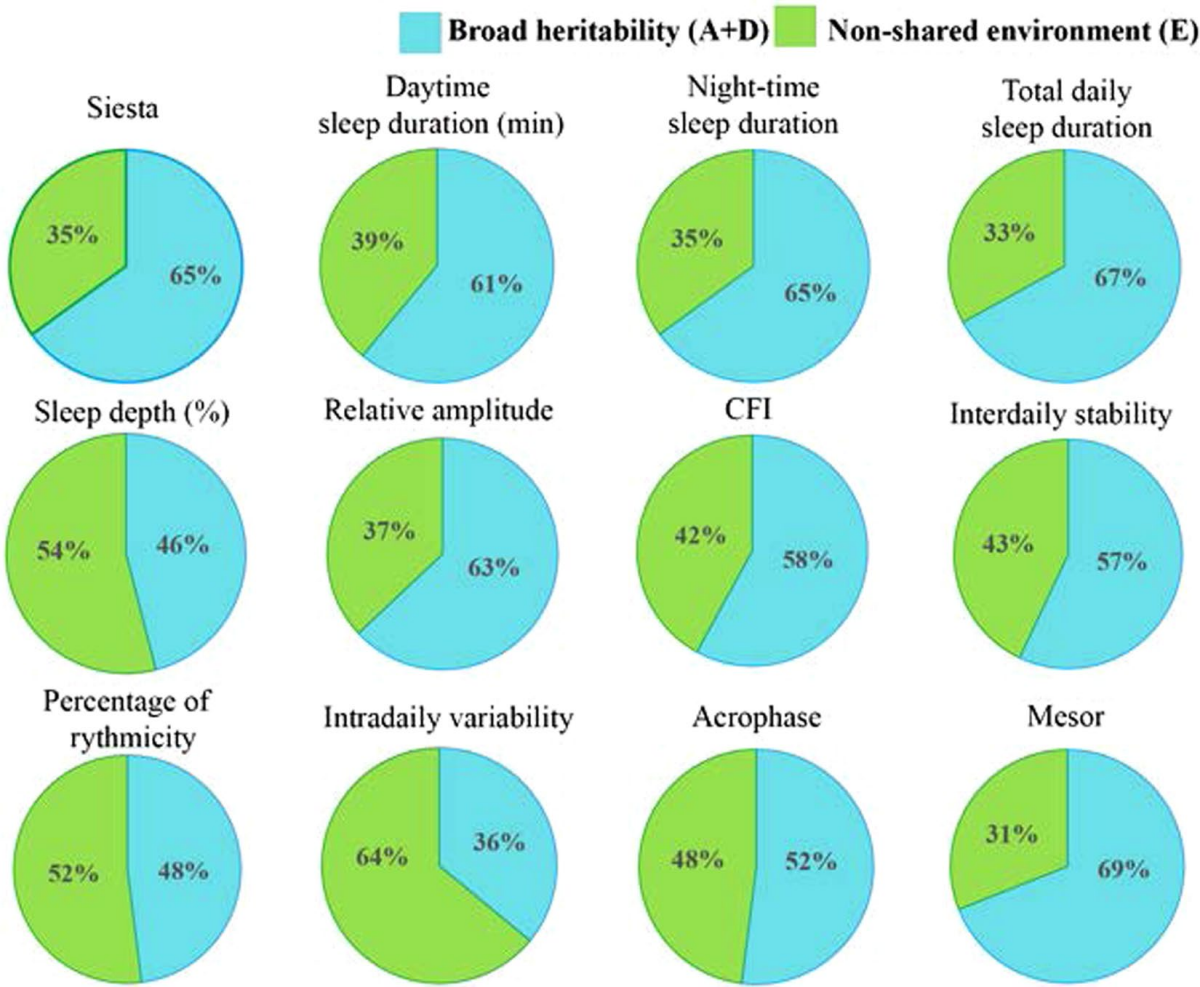
Proportion of variance explained by genetic and environmental factors for the different sleep parameters studied. The pie slices represent the contribution (percentage) of broad heritability (A = additive genetic factor + D = non-additive genetic factors), and non-shared environmental factors (E) to the phenotypic variance of the different variables. CFI: circadian function index. Variables are sorted by function group.

Figure 2 represents the different heritabilities of self-reported siesta and several sleep parameters obtained from the TAP variable such as daytime, night-time and total daily sleep duration, relative amplitude, circadian function index (CFI), interdaily stability, percentage of rhythmicity, intradaily variability, acrophase and mesor. Heritability of taking siesta (self-report) and daytime sleep duration was 65 and 61% respectively. Moreover, heritability of daytime sleep duration was still relevant [A: 45% (95%CI: 13%, 68%); E: 55% (95%CI: 31%, 87%)] after controlling for night-time sleep duration. Similar results were found among the rest of parameters obtained from TAP, with estimated heritability ranging from 36 to 69%, suggesting a relevant genetic impact on sleep rhythm.

## Discussion

This is the first study to investigate the relative contribution of genetic and environmental factors to siesta. By using an integrative variable (TAP) that combines wrist temperature and actigraphy (activity and position), measured during a rather long period of time (7 days), we have been able to analyse the relative contribution of genetic factors to 24h-sleep rhythmicity. The advantage of TAP is that it offered the possibility to assess heritability in free living conditions, without interfering with the subject’s routine. This novel approach provides a unique insight into daytime sleep, and improves our comprehension of the important role played by genetic factors in quality and duration of sleep^12,25,26^.

Our results show a much higher similitude between MZ than DZ twins in taking siesta, as well as in minutes slept during daytime, rendering heritabilities of 65 and 61%, respectively. Noteworthy, the genetic contribution to siesta obtained by these two methods (self-report and TAP) was similar. This genetic effect is reflected in the TAP 24h-pattern, where MZ twins showed both daytime and night-time sleep curves very similar between members of a pair, while DZ twins showed more intra-pair differences.

It is obvious that taking a siesta not only depends on genetic factors but also on cultural habits and environmental conditions; it requires some free time and a convenient space. Our results suggest that, when those conditions are met (as it is the case in this sample), sleepiness following midday meal and the subsequent need to take a nap may be genetically influenced, as well as the length of this rest.

Night-time sleep characteristics and duration are other aspects to consider in the need for taking a nap. It is known that the sleep-wake rhythm is regulated not only by the circadian drive (an internal clock), but also by the homeostatic drive, which increases in case of insufficient sleep in the previous night. Both homeostatic and circadian factors interact with each other^27^. In the current study, heritability of daytime sleep duration was still significant after controlling for night-time sleep duration. This suggests that factors other than a compensatory effect are acting and reinforcing the possible role of genetics in the occurrence of this behaviour.

Aside from daytime sleep characteristics, with the use of TAP, we have been able to investigate quite novel aspects of sleep. For example, the assessment of the sleep pattern continuously during 7 days allowed us to differentiate between “duration of night-time sleep”, with a broad heritability (H^2^) of 65%, and “interdaily stability”, a concept that corresponds to the regularity of such length (H^2^: 57%). Genetic factors that influence whether sleep time is longer or shorter may not be the same as those contributing to the rhythmic character of such length.

Another important aspect of sleep is EEG slow-wave oscillations. Previous studies have shown that the amount of slow-wave sleep points to heritability estimatesof approximately 50%^18^. These data are consistent with our results, which estimate that genetic factors account for 46% of the variance for sleep depth.

Additionally, in this study, TAP provided information over three features of the sleep rhythm: level, timing, and robustness.

Mesor is the “level” parameter related to sleep values (0 is for totally awake and 1 for totally asleep). For this parameter, our data show a heritability of 69%, suggesting a strong genetic influence on the individual variability in this circadian rhythm.

“Timing” of nocturnal sleep, evaluated by cosinor’s acrophase, is one of the most important parameters for sleep characterization and it is related to different pathologies such as the familial advanced sleep phase type, characterized for an extremely early and involuntary sleep timing, or delayed sleep phase type characterized for an inability to fall asleep or awake at a desired time^28^. We previously reported a heritability of 70% for phase of the temperature rhythm^29^, which is closely related to sleep^30^. When integrating different measures in order to obtain a more accurate assessment of sleep rhythm, as in TAP, the heritability estimate appears to decrease (52%) due to the integration of measures with different influences from environmental factors^23^.

Finally, the “robustness” of circadian sleep rhythm is determined by several parameters such as relative amplitude, CFI, interdaily stability, percentage of rhythmicity and intradaily variability. Heritability estimates for these parameters show that genetic factors account for 48 to 63% of their variability, the only exception being intradaily variability, which refers to the fragmentation of sleep and shows a somewhat lower estimate (36%). This parameter may be affected to a greater degree by environmental factors such as light exposure, nocturnal noise, climate conditions or even stress, than other sleep characteristics. Relative amplitude, which determines the changes between maximum sleep and awaking, has been considered as a marker of biological aging^31^. Besides, a robust circadian sleep pattern should be regular and with low fragmentation. These aspects are determined respectively by interdaily stability and intradaily variability. The integration of these three parameters (relative amplitude, interdaily stability and intradaily variability) in the CFI index has an estimated heritability of 58%, suggesting that the robustness of sleep is largely influenced by genetic factors.

In general, our estimates of the relative impact of genetic factors on the characteristics of the sleep pattern, as assessed by TAP, appear to be somewhat higher than those obtained through self-report of sleep features, such as duration or quality. This difference could point to a greater impact of environmental factors in the subjective experience of sleep and suggest that both kind of measures are not directly comparable, what should be taken into account in future studies about sleep rhythms.

In the discussion of our results, we also need to consider some limitations. A larger sample size would have increased the power and would have allowed for more accurate estimation and differentiation between genetic and environmental components of familial factors in specific parameters. This would have also allowed for comparison between sleep rhythms and health-related variables. Additionally, only adult females participated in this study, which limits the conclusions to this gender and age range.

Summarizing, this is the first study to investigate the relative contribution of genetic factors to siesta. By using TAP, we introduce a novel approach to the study of diurnal sleep characteristics. We have reported significant heritabilities related to the sleep rhythm in most parameters, using a methodology that allows for an ambulatory monitoring of objective rhythmic measures. It is noteworthy that this genetic influence appears not only in night-time, but also in diurnal sleep parameters. Despite the above mentioned limitations, our results represent a significant step towards the understanding of novel aspects of the sleep rhythm, such as siesta and the regularity of sleep.

## Materials and Methods

### Subjects

A sample of female twins selected from the Murcia Twin Register (MTR) participated in this study. The MTR is a population-based register of people born in multiple births between 1940 and 1966 in the region of Murcia, southeast Spain. The registry was born in 2006 based on an agreement between the University of Murcia and the Murcia Health Council. Participation in the MTR is voluntary, subjected to informed consent, and not remunerated. Information about the individuals comes from the databases available at the regional health system. As of today, the registry has collected information from 2281 individual twins. More detailed description regarding characteristics and procedures of the MTR can be found elsewhere^32,33^. The MTR management and data collection procedures have been approved by the Committee of Research Ethics of the University of Murcia and it follows national regulations regarding personal data protection. Applicable institutional and governmental regulations concerning the ethical use of human volunteers were followed during this research.

A preliminary procedure selected, from the registry databases, those female pairs living in the same geographical area (within a 30-Km radius from the investigation center) and not suffering from any severe condition that impeded or hindered participation (e.g. cognitive disorders, diabetes mellitus, chronic renal failure, hepatic diseases or cancer). Only pairs with both members meeting such criteria were contacted and offered participation in the current study (n = 118). Finally, a total of 53 pairs of adult female twins volunteered for this study (28 MZ; 25 DZ). Mean age of the selected participants was 52 years old (SD: 6.03; Range: 46–69). Zygosity was determined by DNA testing.

The data analysed in this report were collected between December 2012 and March 2013. An information letter was sent to pre-selected individuals. Later a phone call confirmed their availability, the absence of any condition listed in the exclusion criteria, and their willingness to participate. Participation was completely voluntary and non-remunerated. The subjects were organized in groups of 4–5 pairs, and were given an appointment on a university facility located in the city centre, where informed consent was signed prior to any other procedure.

### Measurement of sleep rhythmicity

Wrist Temperature Rhythmicity. WT rhythm was assessed continuously for 7 days using a temperature sensor (Thermochron iButton DS1921H, Dallas, Maxim, Dallas, TX, USA). The sensor measures 1 to 8 °C increments with ± 1 °C accuracy with a sensitivity of 0.1 °C and programmed to sample every 10 min. It was placed over the inside of the wrist on the radial artery of the non-dominant hand. The information stored in the iButton was transferred through an adapter (DS1402D-DR8; IDC, Spain) to a personal computer using iButton Viewer v. 3.22 (Dallas Semiconductor MAXIM software provided by the manufacturer), as previously described by Sarabia *et al*.^20^.

Body position and rest–activity rhythm. Over those same 7 days, body position and rest–activity rhythms were assessed using a HOBO Pendant G Acceleration Data Logger UA- 004-64 (Onset Computer, Bourne, MA, USA), which was programmed to record data every minute and placed on the non-dominant arm by means of an elastic band, with its X axis parallel to the humerus bone. Two variables were defined from the information provided by the actimeter: motor activity (A) and body position (P)^22^. First, A was calculated as degrees of change in X, Y and Z axis position with respect to the previous sampling time as described by Ortiz-Tudela *et al*.^22^. Then, P was calculated as the angle between X axis of the actimeter and the horizontal plane, with the 0° value being when the arm was in a horizontal position and 90° when it was vertically aligned^22^.

Measurement of TAP rhythmicity. Measures of wrist skin temperature, motor activity and body position were finally integrated into a variable called TAP^24^, according to the Circadianware software implemented in the Kronowizard platform (https://kronowizard.um.es/). Briefly, in order to calculate the integrated TAP variable, we first normalized the temperature, activity and position variables by calculating the 95th and 5th percentiles for each one. Normalized wrist temperature values were inverted since activity and position values were opposites, so that the maximum values for all 3 variables occurred around the same time of the day. Afterwards, we calculated the mean of all 3 normalized variables, where 0 corresponds to complete rest and sleep, and 1 to periods of high arousal and movement^22^. In a subsequent step we calculated an individualized threshold of the frequency distribution of TAP.

#### The following sleep parameters were obtained from registered software (Circadianware®)

- Night-time sleep duration: sleep duration during night from 21:00 to 11:00.
- Daytime sleep duration: minutes of diurnal sleep from 12:00 to 20:00.
- Total daily sleep duration: total sleep duration along 24 hours.
- Sleep depth (%): index of sleep restfulness, determined as the difference between 1 and TAP L5 (average of measurements for the 5 consecutive hours centred in the middle time of sleep). Sleep depth shows its higher values with lower values of TAP. It is expressed in %.

#### Parameters obtained through Cosinor’s analysis

- Mesor: mean value of the rhythm fitted to a cosine function.
- Acrophase: timing of the maximum value of a cosine function.
- Percentage of rhythmicity (PR): percentage of data variance explained by the sinusoidal function. Higher values of this parameter mean to a more sinusoidal curve.

#### Parameters obtained through non-parametric analysis^34^

- Interdaily stability (IS): similarity of the 24 h pattern over days. Its values oscillate between 0 for Gaussian noise and 1 for a perfect stability, where the rhythm repeated itself exactly day after day.
- Intradaily variability (IV): fragmentation of the rhythm. Its values oscillate between 0 for a perfectly sinusoidal wave and 2 for Gaussian noise.
- Relative amplitude (RA): difference between the average of measurements made for the 5 consecutive hours with the maximum sleep (M5) and the average of measurements made for the 10 consecutive hours with the minimum sleep (L10), divided by the sum of both values (M5 + L10).
- Circadian Function Index (CFI): numerical index that determines the circadian robustness based on three circadian parameters: interdaily stability, intradaily variability and relative amplitude^22^. Intradaily variability values were inverted and normalized between 0 and 1, with 0 being a noise signal, and 1 a perfect sinusoid. Finally, CFI was calculated as the average of these 3 parameters performed by the software “Circadianware” implemented in Kronowizard platform (https://kronowizard.um.es/). Consequently, CFI oscillates between 0 (absence of circadian rhythmicity) and 1 (robust circadian rhythm).

Self-reported siesta was recorded as a categorical variable (Yes/No). Subjects that provided daily information about siesta (start and end times) for 1 or more days in a week were considered as siesta-takers, coded as “Yes”; the rest were coded as “No”.

### Other measures

Body weight was valued in barefooted subjects wearing light clothes with the use of a digital scale accurate to the nearest 0.1 Kg. Height was determined using a portable stadiometer (rank, 0.14–2.10). The subjects were positioned upright, relaxed, and with their head in the Frankfort plane. Height and weight measurements were obtained at the beginning of the appointment, all at the same time of day. These data were used to calculate Body Mass Index (BMI) according to the formula: weight (Kg)/ height (m^2^)^35^.

The Horne and Ostberg questionnaire was used to assess the morningness–eveningness^36^. This questionnaire establishes five behavioural categories: definitively morning types (score = 70–86), moderately morning types (score = 59–69), neither types (score = 42–58), moderately evening types (score = 31–41) and definitively evening types (score = 16–30). For the purpose of this study, we reduced the categories from five to three: morning type (score = 59–70), neither type (score = 42–58) and evening type (score = 41–16)^37^.

### Statistical Analyses

Data preparation and descriptive analyses were performed in SPSS v.15.0 (SPSS, 2010). Unpaired group comparisons (t test/Mann-Whitney or χ^2^) were applied to assess mean/percentage difference in selected characteristics between MZ and DZ twins, including morningness-eveningness.

Genetic analyses: Sleep indices that did not adjust to normality were normalized using a rankit procedure previous to analyses^38^. Where present, outliers were excluded from the analysis using three times the interquartile range as reference. Assumptions of the twin design (i.e., equal variances and means for MZ and DZ twins, as well as for co-twins) and possible age effects were tested by comparing twin models to saturated models. No mean or variance differences between twins in a pair or across zygosities were observed. Age effect was not relevant in any case, and it could be dropped from the models with no significant loss of fit.

Next, we tested whether MZ twin intra-pair correlations were higher than DZ twin correlations for each of the phenotypes, which would suggest a genetic influence on individual differences for such trait. Then, genetic influences in the measured parameters were estimated by fitting genetic structural equation models (SEM) in which the observed phenotypic variance is decomposed into genetic and environmental components^39^.

While all sleep parameters obtained from TAP analysis were of a continuous nature, siesta was defined as a categorical variable. It was analysed as such using a liability threshold model. In order to apply variance component genetic models to categorical twin data, it is assumed that categories reflect an imprecise measurement of an underlying normal distribution of liability, which would have one or more thresholds to discriminate between the categories^40^. This liability may be influenced by genetic and environmental factors and it is normally distributed with a mean value of 0 and a variance of 1. Twin similarity can be estimated by the correlation for the liability scale, called tetrachoric correlation.

Observed MZ and DZ twin correlations generally reflect a combination of additive (A; i.e., summed allelic effects across multiple genes) and non-additive (D; i.e., genetic dominance, possibly including epistasis) genetic factors; as well as shared (C; i.e., common/family environment) and individual (E; i.e., idiosyncratic experiences, including measurement error) environmental factors. It is not possible to estimate C and D simultaneously, because C and D are negatively confounded and the choice of modelling C or D depends on the pattern of MZ and DZ correlations. Usually, C is estimated if the DZ twin correlation is greater than half of the MZ twin correlation, and D is estimated if the DZ twin correlation is less than half of the MZ correlation^41,42^.

Structural equation modelling determines the combination that best matches the observed data^43^. Data from the MZ and DZ twin pairs were analysed by using the Open MX software package in R^44^. Overall goodness of fit of the full models (ADE) was determined on the basis of the chi-square (χ^2^) statistic. Subsequently, the significance of genetic factors (A and D) was assessed by means of likelihood ratio tests comparing the full model with a sub-model from which these factors were constrained at zero. When the fit significantly worsened, the contribution of genetic factors was considered significant. The broad heritability (H2) of the phenotype was defined as the percentage of total variance that could be explained by genetic factors (A + D). The power of the experimental design to detect broad sense heritability for different values of H2, based on the current sample size, was determined by testing ADE versus E with a 2 df test and alpha of 5%. The power to detect an H2 of 0.5, 0.6, or 0.8 was 75, 91 and 99% respectively, when the contribution of additive and non-additive effects were equal.
